# Clinical Outcome of a Portosplenomesenteric Venous Thrombosis in Necrotizing Acute Pancreatitis with Protein C and S Deficiency Treated by Anticoagulation Therapy Alone

**DOI:** 10.1155/2015/934784

**Published:** 2015-09-14

**Authors:** Firmin Ankouane, Mathurin Kowo, Bernadette Ngo Nonga, Eric Magny, Edith Hell Medjo, Elie Claude Ndjitoyap Ndam

**Affiliations:** ^1^Department of Internal Medicine and Specialties, Faculty of Medicine and Biomedical Sciences, University of Yaounde 1, Yaounde University Teaching Hospital, Yaoundé, Cameroon; ^2^Department of Surgery and Specialties, Faculty of Medicine and Biomedical Sciences, University of Yaounde 1, Yaounde University Teaching Hospital, Yaoundé, Cameroon; ^3^Department of Radiology and Imaging, Faculty of Medicine and Biomedical Sciences, University of Yaounde 1, Yaoundé, Cameroon; ^4^Department of Internal Medicine and Specialties, Faculty of Medicine and Biomedical Sciences, University of Yaounde 1, Yaounde General Hospital, Yaoundé, Cameroon

## Abstract

Cases of splanchnic venous thrombosis have not been described in Cameroon. Their prevalence in acute pancreatitis is variable. With the emergence of acute intra-abdominal infections including typhoid fever and peritoneal tuberculosis in situations of acquired immunodeficiency syndrome, these cases will become frequent. We report the case of a portosplenomesenteric venous thrombosis related to necrotizing acute pancreatitis associated with proteins C and S deficiency, in a 46-year-old Cameroonian man, without particular past medical history. He was admitted for abdominal pain which had been evolving for 3 weeks and accompanied by vomiting. In the absence of hemorrhagic risk factor, the patient received low molecular weight heparin followed by oral warfarin. The abdominal ultrasound check on the 12th day showed a partial recanalization of venous thrombosis. The abdominal contrast-enhanced CT scanner at day 30 on oral anticoagulation therapy showed collateral vessels and small bowel edema. At the same time the upper gastrointestinal endoscopy showed grade II esophageal varices. We have maintained oral anticoagulation therapy. This case highlights that an early effective anticoagulation heparin therapy is needed for a clear benefit in case of suspected PSMVT. It is certain that the sooner the treatment is given, the better outcome will be.

## 1. Introduction

Venous thrombosis is a multifactorial disease resulting from the dynamic interaction between genetic and acquired risk factors. The common inflammatory causes of splanchnic venous thrombosis (SVT) are acute abdominal pathologies [1]. The portosplenomesenteric venous thrombosis (PSMVT) is rare [2]. In acute pancreatitis (AP) it has been found to be related to the severity of the pancreatitis. Necrotizing AP is accompanied by perivascular inflammation, pancreatic necrosis, and compression by peripancreatic collections or pseudocysts as well as protein C and S deficiency [2–4].

The use of unfractionated heparin or low molecular weight heparin followed by oral vitamin K antagonists is the common approach to anticoagulation [2]. The complications directly related to SVT are rare but PSMVT is associated with a more dismal prognosis [5]. We are reporting here the first case of PSMVT complicating a necrotizing pancreatitis which was associated with protein C and S deficiency.

## 2. Case Presentation

**Figure 1:**
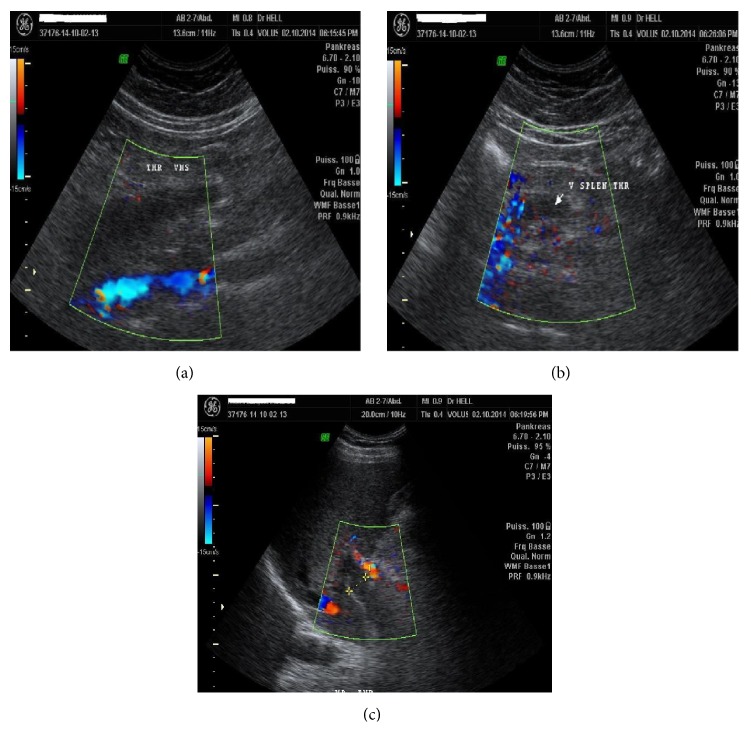
Doppler ultrasonography: showing in (a) the superior mesenteric vein thrombosis, in (b) the splenic vein thrombosis, and in (c) the portal vein thrombosis. With the permission of Medical Imaging Centre (MIC) in Yaoundé.

**Figure 2:**
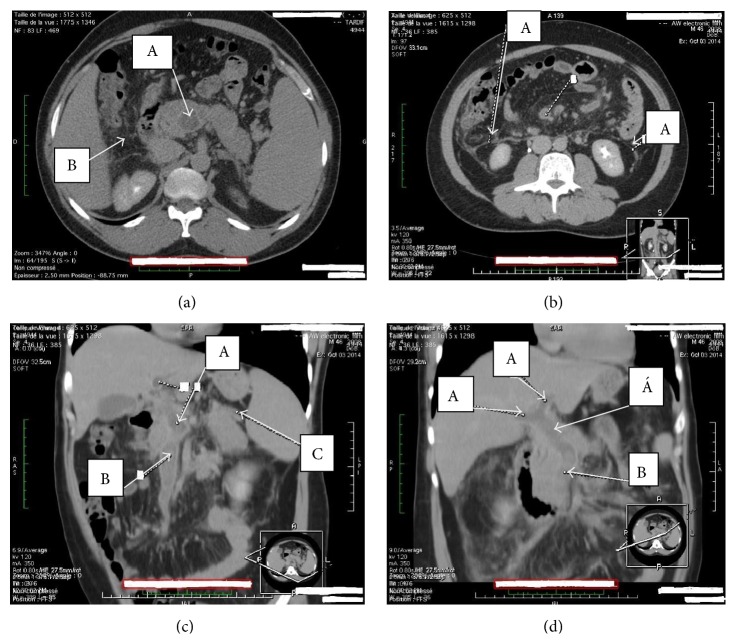
Abdominal contrast-enhanced CT scanner (hospital admission): (a) an edematous pancreas in (A) the superior mesenteric vein thrombosis and (B) the infiltration of the peripancreatic fat. (b) (A) Inflammatory flows. (c) (A) The portal venous thrombosis, (B) the superior mesenteric venous thrombosis, and (C) the splenic venous thrombosis. (d) (A) The thrombosis of the left and right branches of the portal vein and (B) the superior mesenteric venous thrombosis and distal branches. Light Speed CT-16 (GE Medical Systems, USA). Contrast: iobitridol 300 mg/mL (Xenetix, Guerbet, Roissy CDG Cedex, France). With the permission of the Cathedral Medical Center (CMC), Yaoundé.

A 46-year-old Cameroonian man, living in North Cameroon, was admitted to the Yaoundé University Teaching Hospital for diffuse abdominal pain accompanied by abdominal distension and vomiting for three-week duration. This was treated as a gastroduodenal ulcer without accurate diagnosis on endoscopy. His past medical history was unremarkable. The physical exam revealed a distended and tympanic abdomen, diffusely tender with no guarding. There was no palpable mass and bowel sounds were present. The digital rectal exam elicited pains on both sides. On admission, he has a low grade fever with a temperature of 38.2°C; the blood pressure was 113/82 mm Hg, and the heart rate 102 beats/minute. The laboratory tests (hemogram and biochemistry) were normal except for a C-reactive protein of 160 mg/L (standards: less than 6 mg/L), the lipase of 6xULN (upper limit of normal), and mild hepatic cytolysis (ALT greater than 3xULN). The tests of the hepatitis B surface antigen, the anti-hepatitis C virus antibodies, and the human immunodeficiency virus (HIV) were negative. Abdominal Doppler ultrasonography revealed an extensive venous thrombosis of the portal, splenic, and mesenteric veins (Figure 1). The abdominal contrast-enhanced CT scanner confirmed the extended thrombosis and highlighted an infiltration of the proximal pancreas associated with inflammatory flows in the peritoneum (Figure 2).

**Figure 3:**
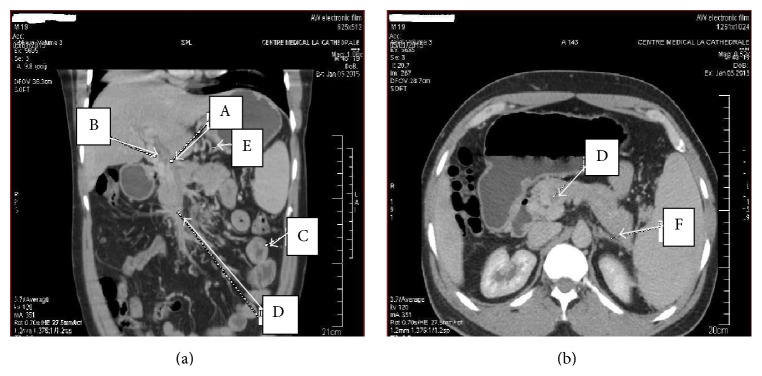
Abdominal contrast-enhanced CT scanner: control after three months on oral anticoagulation. (a) Partial recanalization of the portal venous thrombosis (A) and the superior mesenteric venous thrombosis (D), the portal cavernoma (B) and gastric collateral vessels (E), and thickening of the small intestinal bowel (C). (b) Superior mesenteric venous partial recanalization (D) and residual inflammatory flows (F). Light Speed CT-16 (GE Medical Systems, USA), contrast: iobitridol 300 mg/mL (Xenetix, Guerbet, Roissy CDG Cedex, France). With the permission of the Cathedral Medical Center (CMC), Yaoundé.

**Figure 4:**
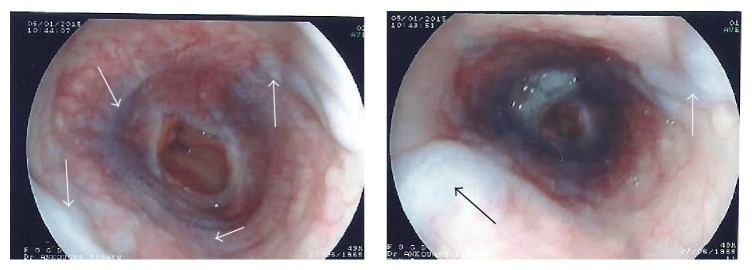
Upper gastrointestinal endoscopy performed after three months on oral anticoagulation. Arrows show medium varices occupying the circumference of the esophageal lumen. Fujinon EG250PE5, Japan. With the permission of the Cathedral Medical Center (CMC) in Yaoundé.

The diagnosis of necrotizing AP complicated by PSMVT was done. In the absence of significant contraindications factor, the patient received low molecular weight heparin (enoxaparin 1 mg/kg subcutaneously twice daily), followed by oral warfarin on the 10th day, dose adjusted according to the International Normalized Ratio (INR). The repeated abdominal Doppler ultrasound on the 12th day from admission showed a partial recanalization of the extensive venous thrombosis, confirmed by the abdominal contrast-enhanced CT scanner on 30th day which showed collateral vessels and small bowel edema (Figure 3). For accurate diagnosis, specific tests have shown normal serum lipids, and the tests for anti-nuclear antibodies, anti-native DNA antibodies, and antiphospholipids were negative; the hemoglobin electrophoresis was AA type. On the contrary, the functional test of protein C (STA Stachrom) showed a serum rate of 25% (normal range: 80–130%, Cerba Laboratories, France) and that of the protein S (STA Staclot) was 50% (normal range: 70–130%, Cerba Laboratories, France). Upper gastrointestinal endoscopy performed three months after follow-up showed grade II esophageal varices (Figure 4). There were no adverse effects on anticoagulation after three months of follow-up.

## 3. Discussion

Vascular complications have been associated with AP and these include SVT (1-2%) [5]. The different causes for splanchnic vein thrombosis are well established in the following list.

*Known Causes of Splanchnic Vein Thrombosis*

*(1) Abdominal disorders and postoperative state are as follows:*

(a) acute:
  (i) pancreatitis^*∗*^,
  (ii) peritonitis and intra-abdominal sepsis^*∗*^,
  (iii) inflammatory bowel disease,
  (iv) diverticulitis,
  (v) splenectomy,
  (vi) abdominal operations^*∗*^,
  (vii) sclerotherapy for esophageal varices,
  (viii) blunt abdominal trauma;
(b) chronic:
  (i) cirrhosis and portal hypertension,
  (ii) neoplasm^*∗*^.

*(2) Prothrombotic states are as follows:*

- Antithrombin III deficiency.
- Protein C deficiency.
- Protein S deficiency.
- Factor V Leiden.
- G20210A mutation in prothrombin gene.
- Antiphospholipid antibodies.
- Hyperhomocysteinemia.

*(3) Hematologic disorders are as follows:*

(i) Polycythemia vera^*∗*^.
(ii) Essential thrombocythemia^*∗*^.
(iii) Idiopathic myelofibrosis.
(iv) Paroxysmal nocturnal hemoglobinuria^*∗*^ (PNH).

*(4) Hormones are as follows:*

(i) Oral contraceptives^*∗*^.
(ii) Hormonal replacement therapy.
(iii) Pregnancy.
(iv) Puerperium.

*(5) Autoimmune disorders are as follows:*

- Behçet's disease.
- Hypereosinophilic syndrome.

*(6) Miscellaneous causes are as follows:*

- Decompression sickness.
- Cytomegalovirus.

*(7) New biological markers of subclinical disorders are as follows:*

- JAK2 mutation.
- CD55 and CD59 (PNH clone).

^*∗*^This factor is among the more common causes of splanchnic vein thrombosis.

Among these, the splenic venous thrombosis is the most common due to its proximity with the pancreas, far ahead of portal venous thrombosis and the superior mesenteric venous thrombosis [3, 5, 11–13]. Portosplenomesenteric venous thrombosis is very rare and it is extremely variable; this is the first case of PSMVT associated with AP reported from Cameroon; both diseases are very rare in our environment and have not been reported in the literature. For example, in a two-year prospective study, Gonzelez et al. [2] reported one case out of 127 patients admitted for AP.

In the context of the AP, inflammatory phenomena largely explain the occurrence of thrombosis. They include local prothrombotic intraendothelial phenomena [2, 6]. In the majority of cases, the development of PSMVT is associated with the presence, location, and extension of the pancreatic necrosis [2, 3].

Systemic activation of the coagulation system commonly occurs in over half of the critically ill patients with severe AP, and coagulation disorders are related to the severity of the disease [4]. Thus, they frequently include a deficit in proteins C and S [4]. The protein C and S deficiency, which are natural anticoagulants in blood and have an essential role in the regulation of the coagulation, are a known cause of SVT [7]. The AP of our observation was associated with deficiency in proteins C (25%) and S (50%) and inflammatory phenomena. Previous human studies have shown low activity of protein C in severe AP [4, 8]. Evaluation of the coagulation and the endogenous protein C/Antithrombin III system shows that nonsurvivors in severe AP have significantly lower levels of protein C and Antithrombin III than survivors [4, 9, 10]. Also, in severe AP, protein C pathway defects have been shown to be associated with development of organ failure [10]. In our case, a repeat assay on day 90 after presentation shows that protein C and S values were within their normal ranges, which suggests that this deficiency was acquired, due to AP. In our milieu, the prevalence of protein C and S deficiency in a healthy population is unknown. We believe that AP preceded the onset of PSMVT and protein C and S deficiency is a result of the pancreatic necrosis. Diverticulitis is another possible cause of SVT in this patient. However, abdominal CT scanner showed diverticula of the colon without inflammatory signs on them.

It has been reported that SVT can lead to bleeding, bowel ischemia, and liver failure, but the signs and symptoms may overlap with those of AP [2, 11, 12]. Thus, in this case, it was difficult to distinguish between symptoms related to the AP and those related to venous thrombosis.

The combination of Doppler ultrasonography and CT scanner increases the diagnostic sensitivity. Doppler ultrasonography and CT scanner have a negative predictive value of 98% and 100%, respectively. Both tests enable showing pseudoaneurysm, intrapseudocystic bleeding, intra-abdominal collections, venous thrombosis, and varices [11, 12]. We performed both exams for diagnosis in this case.

There is no randomized study related to the use of anticoagulants in acute SVT. The use of unfractionated heparin or low molecular weight heparin during hospital stay followed by oral vitamin K antagonists is the common approach to anticoagulation [13]. The doses of anticoagulation treatment and its duration are not well defined [13]. Whatever the type of treatment used, full recanalization is rare. It has been found only in 1/3 of cases, especially in the recently formed thrombosis [2, 3, 13, 14]. The percentage of that recanalization depends on how early the anticoagulant therapy is given [2, 13, 15]. We started the anticoagulation treatment 3 weeks after the onset of symptoms and we observed a partial recanalization on the 12th day of treatment. The delay of treatment in relation with the installation of thrombosis is difficult to specify in this case. This partial recanalization is probably related to a delay in starting the treatment.

Other invasive approaches such as thrombolysis and transjugular intrahepatic portosystemic stent-shunt (TIPS) exist [16]. Surgical thrombectomy has been reported, especially in patients with high risk of complications to anticoagulation and thrombolytic treatment [17].

Apart from visceral ischemia, PSMVT may be accompanied by hemorrhage. The incidence of this complication is not well defined. In a previous retrospective study concerning 53 patients with AP, Heider et al. [18] reported that gastric variceal bleeding from AP-induced SVT occurs in only 4% of the cases. The majority of patients with PSMVT develop collateral vessels and more than a quarter presented varices on endoscopy [3, 19]. This case developed collateral vessels, esophageal varices, and small bowel edema on anticoagulation three months following abdominal complaints onset.

In conclusion, we report a clinical outcome of PSMVT in necrotizing AP with combined deficiency of proteins C and S in 46-year-old Cameroonian man treated with anticoagulation alone. This case highlights that an early effective anticoagulation heparin therapy is needed for a clear benefit in case of suspected PSMVT. It is certain that the sooner the treatment is given, the better the outcome will be.
